# Glucose-regulated protein 75 determines ER–mitochondrial coupling and sensitivity to oxidative stress in neuronal cells

**DOI:** 10.1038/cddiscovery.2017.76

**Published:** 2017-11-06

**Authors:** Birgit Honrath, Isabell Metz, Nadia Bendridi, Jennifer Rieusset, Carsten Culmsee, Amalia M Dolga

**Affiliations:** 1 Institute of Pharmacology and Clinical Pharmacy, University of Marburg, Marburg, Germany; 2 Faculty of Science and Engineering, Groningen Research Institute of Pharmacy (GRIP), Department of Molecular Pharmacology, University of Groningen, Groningen, The Netherlands; 3 Laboratoire CarMeN, INSERM U1060, INRA U1235, Lyon University, Université Claude Bernard Lyon1, INSA-Lyon, Oullins, France

## Abstract

The crosstalk between different organelles allows for the exchange of proteins, lipids and ions. Endoplasmic reticulum (ER) and mitochondria are physically linked and signal through the mitochondria-associated membrane (MAM) to regulate the transfer of Ca^2+^ from ER stores into the mitochondrial matrix, thereby affecting mitochondrial function and intracellular Ca^2+^ homeostasis. The chaperone glucose-regulated protein 75 (GRP75) is a key protein expressed at the MAM interface which regulates ER–mitochondrial Ca^2+^ transfer. Previous studies revealed that modulation of GRP75 expression largely affected mitochondrial integrity and vulnerability to cell death. In the present study, we show that genetic ablation of GRP75, by weakening ER–mitochondrial junctions, provided protection against mitochondrial dysfunction and cell death in a model of glutamate-induced oxidative stress. Interestingly, GRP75 silencing attenuated both cytosolic and mitochondrial Ca^2+^ overload in conditions of oxidative stress, blocked the formation of reactive oxygen species and preserved mitochondrial respiration. These data revealed a major role for GRP75 in regulating mitochondrial function, Ca^2+^ and redox homeostasis. In line, GRP75 overexpression enhanced oxidative cell death induced by glutamate. Overall, our findings suggest weakening ER–mitochondrial connectivity by GRP75 inhibition as a novel protective approach in paradigms of oxidative stress in neuronal cells.

## Introduction

Maintaining intracellular Ca^2+^ ([Ca^2+^]_i_) homeostasis is of major importance to preserve cell survival in neuronal tissues, as for instance oxidative stress induces massive Ca^2+^ influx through different receptor-operated or voltage-dependent Ca^2+^ channels.^1,2^ Enhanced Ca^2+^ influx together with Ca^2+^ release from internal stores such as the endoplasmic reticulum (ER) leads to mitochondrial Ca^2+^ overload and cell death.^3–6^

Small Ca^2+^ microdomains are frequently transferred from the ER to the mitochondria as part of homeostatic organelle communication.^7–9^ The propagation of these Ca^2+^ microdomains is regulated by a multiprotein complex formed by voltage-dependent anion channel 1 (VDAC1) located at the outer mitochondrial membrane, the inositol-1,4,5-trisphosphate receptor (IP_3_R) on the ER membrane and glucose-regulated protein 75 (GRP75), a member of the heat shock protein 70 family.^10–14^ Proper integration of this multiprotein complex into the mitochondria-associated membrane (MAM) is critical for Ca^2+^ transfer into the mitochondrial matrix via the tightly regulated mitochondrial Ca^2+^ uniporter which drives mitochondrial metabolism.^15–19^

By establishing local contact points between ER and mitochondria, GRP75 has a major role in maintaining crosstalk between these organelles through coordinating the exchange and transfer of Ca^2+^, and to drive subsequent signaling cascades.^11,20–23^ GRP75 has been extensively studied in various cancer cells where its expression increased susceptibility to cell death.^24,25^ However, the consequences of an alteration in GRP75 expression to neuronal cell survival are not entirely clear. For instance, GRP75 overexpression in SH-SY5Y cells reduced basal levels of reactive oxygen species (ROS) in physiological conditions, and GRP75 knockdown in these cells activated mitochondrial stress responses. However, following proteolytic stress initiated by overexpression of mitochondrial ornithine transcarbamylase, stress-induced ROS formation and loss of the mitochondrial membrane potential (MMP) was prevented by GRP75 overexpression.^26,27^ In contrast, GRP75 overexpression in dopaminergic neurons exposed to the mitochondrial complex I inhibitor rotenone enhanced cell death, and overexpression of GRP75 in rat mesencephalic neuronal cells potentiated the effects of rotenone on mitochondrial complex I inhibition and oxidative stress.^28^ These studies suggest that GRP75 might mediate both beneficial or harmful effects depending on the cell type, and the pathological context. Thus, further studies are required to clarify the function of GRP75 in paradigms of cell death relevant to neurodegenerative diseases.

In the present study, we sought to investigate the impact of GRP75 expression in neuronal HT22 cells in conditions of oxidative stress and mitochondrial dysfunction. In these immortalized hippocampal neurons, exposure to high concentrations of extracellular glutamate induces oxidative stress and a form of cell death termed oxytosis.^29^ Glutamate-induced oxytosis involves severe mitochondrial damage through loss of MMP, accumulation of ROS and massive influx of extracellular Ca^2+^ along with extensive mitochondrial fragmentation.^30–33^ Preventing mitochondrial dysfunction using different strategies such as activation of Ca^2+^-activated K^+^ channels, inhibition of lipoxygenases, suppressing the mitochondrial translocation of pro-apoptotic Bid or nuclear translocation of apoptosis-inducing factor AIF successfully blocked glutamate-induced cell death in HT22 cells.^31,34–36^ However, the role of GRP75 and organelle crosstalk in this mitochondrial death pathway is so far unknown. In our study, we analyzed the impact of altered GRP75 expression on mitochondrial function and cell death. We show for the first time that silencing GRP75 expression impaired ER–mitochondrial coupling and enhanced mitochondrial resilience in a neuronal model of oxidative cell death.

## Results

### GRP75 determines ER–mitochondrial coupling in neuronal HT22 cells

GRP75 creates a physical link between the ER membrane and the outer mitochondrial membrane through facilitating the interaction between ER-bound IP_3_R and mitochondrial VDAC1.^10,11^

**Figure 1:**
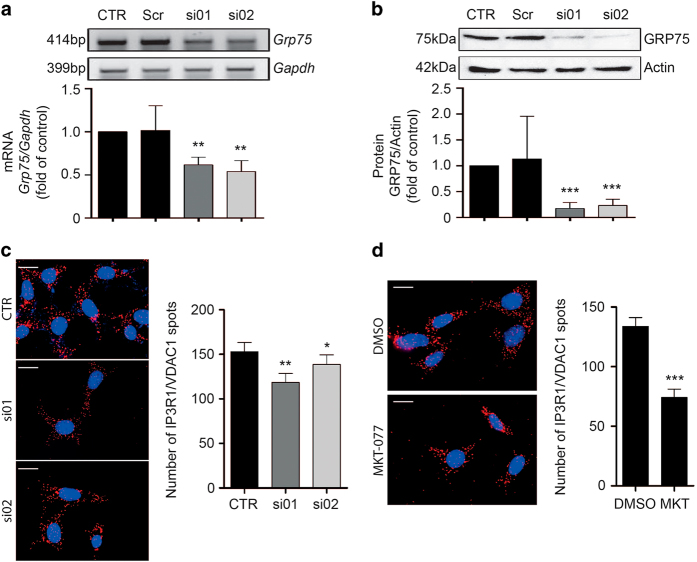
ER–mitochondrial contact points are established by GRP75. (**a**) mRNA levels of *Grp75* and *Gapdh* in CTR, cells transfected with scrambled siRNA (Scr) and cells transfected with siRNAs against GRP75 (si01, si02). Knockdown on mRNA level is quantified by densitometric analysis. Data are presented as mean + S.D., *n*=3, unpaired Student’s *t*-test, **P<0.01. (**b**) GRP75 protein expression in CTR, cells transfected with Scr and cells transfected with siRNAs against GRP75 (si01, si02). Actin is used as a loading control. Knockdown on protein level is quantified by densitometric analysis. Data are presented as mean + S.D., *n*=5, unpaired Student’s *t*-test, ***P<0.0001. (**c**) *In situ* proximity ligation assay (PLA) in CTR, cells transfected with Scr and cells transfected with siRNAs against GRP75 (si01, si02). Left panels: representative images of HT22 cells after *in situ* PLA, DAPI-stained nuclei. Scale bar: 20 *μ*m. Right panel: quantification of IP_3_R/VDAC1 spots on the analyzed pictures. Data are presented as mean + S.E.M., *n*=25–30 per condition, *P<0.05. (**d**) *In situ* PLA in HT22 cells treated with DMSO as a control or with 10 *μ*M MKT-077. Left panels: representative images of HT22 cells after *in situ* PLA, DAPI-stained nuclei. Scale bar: 20 *μ*m. Right panel: quantification of IP_3_R/VDAC1 spots on the analyzed pictures. Data are presented as mean + S.E.M., *n*=25–30 per condition. CTR, control cells.

To confirm that GRP75 is involved in MAM formation in neuronal HT22 cells, we applied two different small interfering RNA (siRNA) sequences targeting GRP75 and performed an *in situ* proximity ligation assay (*in situ* PLA) to assess the interaction between IP_3_R1 and VDAC1. Successful gene silencing of GRP75 successfully was confirmed at the level of mRNA (Figure 1a) and protein expression (Figure 1b). Following GRP75 silencing, we found that IP_3_R1–VDAC1 interaction sites, as indicated by the red punctae (Figure 1c), were reduced compared with control cells.

To validate these findings, we also analyzed IP_3_R1–VDAC1 interaction sites following the application of the pharmacological GRP75 inhibitor MKT-077. Treatment with 10 *μ*M MKT-077, an HSP70 inhibitor known to inhibit GRP75,^37–39^ also reduced the number of red punctae compared with DMSO-treated control cells (Figure 1d) indicating that both pharmacological inhibition and GRP75 depletion reduced ER–mitochondrial contact formation in HT22 cells.

### GRP75 downregulation prevents glutamate-induced cell death

Exposure of neuronal HT22 cells to toxic glutamate concentrations initiates a signaling cascade that mediates cell death through excessive production of ROS and destruction of mitochondria.^31^

**Figure 2:**
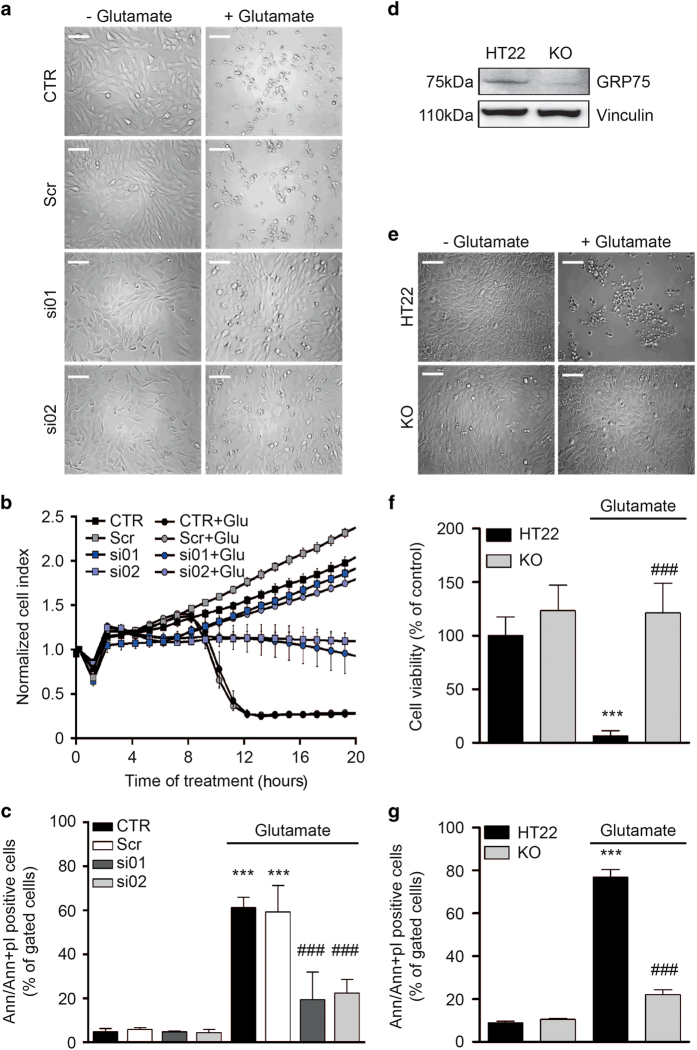
Silencing of GRP75 protects against glutamate-induced cell death. (**a**) Light microscopic pictures of CTR, cells transfected with scrambled siRNA (Scr) and cells transfected with siRNAs against GRP75 (si01, si02) in the absence (left panel) or presence (right panel) of glutamate (16 h). Scale bar: 30 *μ*m, 20× magnification, *n*=3. (**b**) xCELLigence measurement of cell viability following glutamate treatment of CTR, cells transfected with Scr and cells transfected with siRNAs against GRP75 (si01, si02). Data are presented as mean ± S.D., *n*=6–8 per condition. (**c**) Annexin V (early apoptosis) and propidium iodide (late apoptosis) double staining of CTR and cells transfected with Scr or with siRNAs against GRP75 (si01, si02) following glutamate exposure for 16 h. Data are presented as mean + S.D., *n*=3, unpaired Student’s *t*-test, ***P<0.0001 compared with control, ^###^*P*>0.0001 compared with glutamate. (**d**) Western blot analysis of HT22 CTR and GRP75 KO cells showing GRP75 (75 kDa) expression and vinculin (110 kDa) as a loading control (*n*=3). (**e**) Light microscopic pictures of HT22 cells and GRP75 KO cells 16 h following the glutamate challenge. Scale bar: 30 *μ*m, 20× magnification, *n*=3. (**f**) Cell viability analysis using the MTT Assay in HT22 cells and GRP75 KO cells following the glutamate challenge (16 h). Data are presented as mean + S.D., *n*=8. (**g**) Annexin V (early apoptosis) and propidium iodide (late apoptosis) double staining of HT22 and GRP75 KO following glutamate exposure for 16 h. Data are presented as mean + S.D., *n*=3, unpaired Student’s *t*-test, ***P<0.0001 compared with control, ^###^*P*>0.0001 compared with glutamate. CTR, control cells.

In order to investigate whether impaired ER–mitochondrial contact formation could alter cell death signaling induced by oxidative stress, we downregulated GRP75 expression by two different siRNA sequences followed by initiation of cell death with glutamate. Indeed, glutamate induced morphological signs of cell damage; the HT22 cells rounded up and detached from the culture dish (Figure 2a). This was largely prevented in cells that were transfected with the siRNAs before the glutamate challenge. An impedance-based measurement of cell viability^40^ revealed that siRNA-mediated GRP75 silencing fully blocked glutamate-induced cell death (Figure 2b). This was further confirmed by fluorescence-activated cell sorting analysis of early and late apoptotic cells using annexin V (AV) and propidium iodide (PI) double staining. The glutamate-induced increase in AV and AV/PI positive cells was prevented by knockdown of GRP75 (Figure 2c). Importantly, GRP75 knockdown in the absence of glutamate did not cause changes in morphology, proliferation or cell viability.

To validate these findings at the genetic level, we generated a knockout (KO) cell line of GRP75 using the CRISPR/Cas9 technique. Western blot analysis showed that there was no protein expression of GRP75 in the KO colony (Figure 2d). In line with the previous results obtained with siRNA-mediated gene silencing, GRP75 KO cells were protected against glutamate-induced oxidative cell death as shown at the morphological level (Figure 2e), by the MTT Assay (Figure 2f) and by AV/PI double staining (Figure 2g). Similar to GRP75 knockdown, also genetic KO of GRP75 did not change the cellular morphology, affect proliferation or induce cell death under basal conditions.

**Figure 3:**
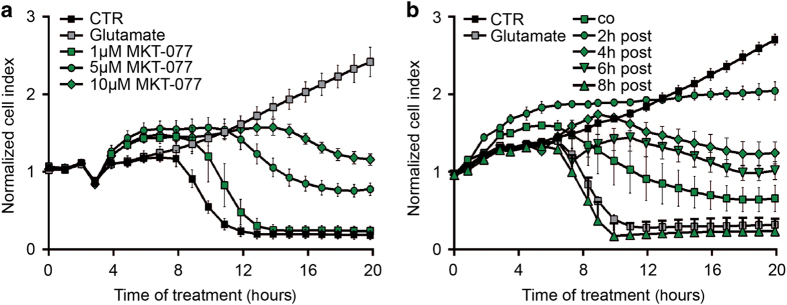
Inhibition of GRP75 prevents glutamate-induced cell death. (**a**) xCELLigence measurement of HT22 cells treated with glutamate and different concentrations of MKT-077 (1, 5, 10 *μ*M). Data are presented as mean ± S.D., *n*=6–8 per condition. (**b**) xCELLigence measurement of HT22 cells treated with glutamate and 10 *μ*M MKT-077. MKT-077 was applied together with glutamate or 2–8 h following glutamate exposure (post-treatment). Data are presented as mean ± S.D., *n*=6–8 per condition.

Further, we applied MKT-077 (10 *μ*M) which downregulated the IP_3_R–VDAC1 interaction as assessed by *in situ* PLA, and tested for neuroprotective effects against glutamate toxicity. MKT-077 preserved cell viability in a dose-dependent manner (Figure 3a) as indicated by real-time cellular impedance measurements in conditions of glutamate toxicity. Furthermore, MKT-077 (10 *μ*M) provided protection against cell death, even when it was applied up to 6 h after onset of the glutamate exposure (Figure 3b).

**Figure 4:**
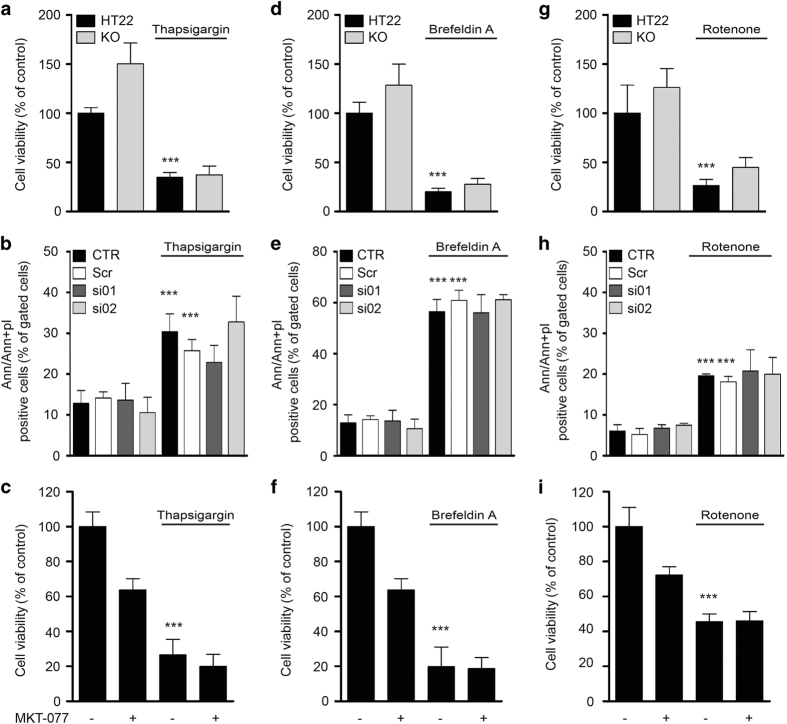
Knockdown, KO and inhibition of GRP75 fails to protect in other paradigms of cell death. Cell viability analysis using the MTT assay (**a**, **d**, **g**) of HT22 and GRP75 KO cells, (**b**, **e**, **h**) of HT22 CTR and cells transfected with scrambled siRNA (Scr) or siRNA against GRP75 (si01, si02), or (**c**, **f**, **i**) of HT22 cells treated with 10 *μ*M MKT-077 following exposure to (**a**–**c**) 2 *μ*M thapsigargin, (**d**–**f**) 2 *μ*M brefeldin A or (**g**–**i**) 50 *μ*M rotenone for 24 h or 16 h, respectively. Data are presented as mean+S.D., *n*=8 per condition, unpaired Student’s *t*-test, ***P<0.0001. CTR, control cells.

Enforcing the physical linkage between ER and mitochondria increases mitochondrial Ca^2+^ uptake, thus susceptibility to stress.^41^ To test whether the impairment of ER–mitochondrial coupling also protected against ER stress, we exposed KO cells, GRP75-silenced cells, cells treated with MKT-077 (10 *μ*M) and respective controls to ER stress for 24 h. ER stress was induced by thapsigargin, a sarcoplasmic/endoplasmic reticulum Ca^2+^ ATPase (SERCA) inhibitor^42^ or by brefeldin A that leads to accumulation of unfolded proteins in the ER lumen.^43^ Neither KO, knockdown, nor pharmacological inhibition of GRP75 by MKT-077 could prevent cell death induced by thapsigargin (Figures 4a–c) or brefeldin A (Figures 4d–f) as assessed by the MTT assay and AV/PI double staining. In addition, we tested whether downregulation of GRP75 rescued HT22 cells from cytotoxicity induced by the mitochondrial complex I inhibitor rotenone.^44,45^ AV/PI double staining and the MTT assay revealed that neither GRP75 KO, GRP75 siRNA, nor pharmacological inhibition by MKT-077 conferred protection in this paradigm of rotenone-induced cell death (Figures 4g–i).

In summary, genetic downregulation of GRP75 prevented glutamate-induced oxidative cell death but failed to protect against ER stress or mitochondrial complex I inhibition. Thus, the ER–mitochondrial junction seems to play a critical role in cell death cascades where mitochondrial damage is a consequence of oxidative stress initiated upstream of mitochondria, while it is dispensable for ER stress and for toxicity caused by direct mitochondrial damage.

### GRP75 depletion preserves mitochondrial function and regulates [Ca^2+^]_i_ homeostasis

Mitochondrial fragmentation, loss of mitochondrial function and mitochondrial demise are major hallmarks of oxidative glutamate toxicity in neuronal cells, and strategies for preserving mitochondrial function are widely investigated due to their promising therapeutic potential in neurological diseases. Here, we exploited the neuroprotective potential of diminishing ER–mitochondrial contact formation on mitochondrial integrity during the oxidative glutamate challenge.

**Figure 5:**
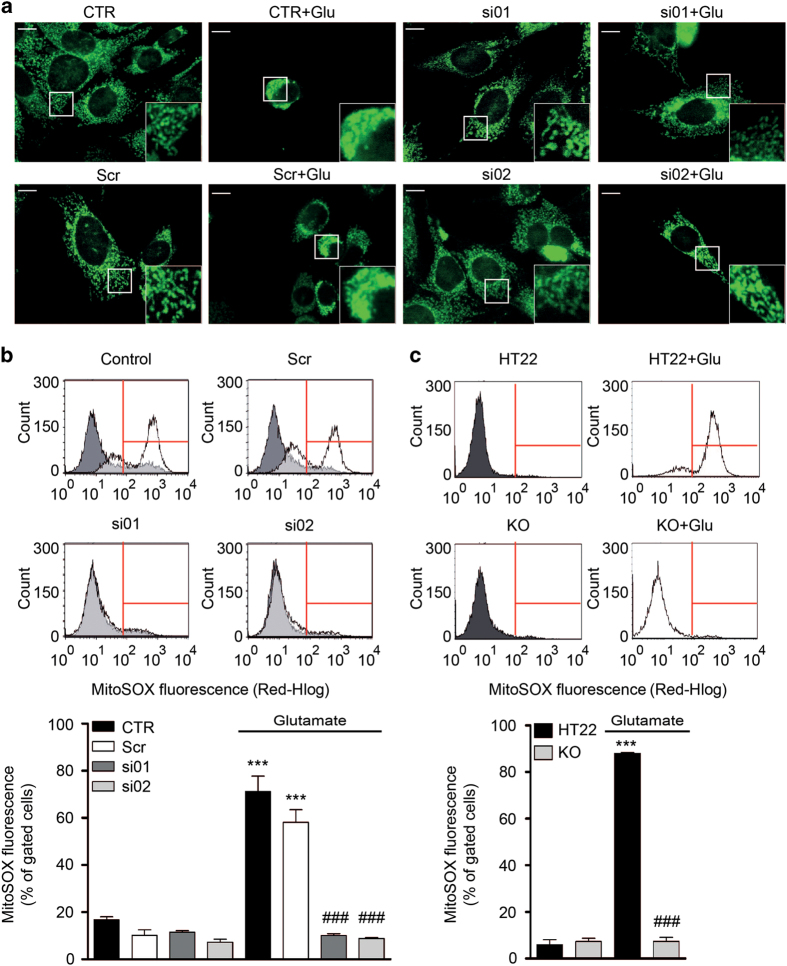
Ablating GRP75 preserves mitochondrial morphology and attenuates mitochondrial ROS formation in response to glutamate treatment. (**a**) Representative images of mitochondrial morphology in HT22 CTR and cells transfected with scrambled siRNA (Scr) or siRNA against GRP75 (si01, si02) in the presence or absence of glutamate (16 h). Mitochondria are stained with MitoTracker Green. Scale bar: 10 *μ*m, 63× magnification, *n*=3. (**b**, **c**) Representative measurement of mitochondrial ROS following glutamate exposure (16 h) in (**b**) HT22 CTR and cells transfected with scrambled siRNA (Scr) or siRNA against GRP75 (si01, si02), and (**c**) HT22 and GRP75 KO cells. Data are presented as mean + S.D., *n*=3, ***P<0.0001 compared with control, ^###^*P*<0.0001 compared with glutamate. CTR, control cells.

First, we analyzed the mitochondrial morphology after glutamate exposure and classified cells into three categories (category I: cells containing elongated, widely distributed mitochondria; category II: cells containing elongated and fragmented mitochondria, widely distributed in the cell; category III: cells with fragmented mitochondria, accumulated around the nucleus).^32^ Fluorescence imaging and quantification of the mitochondrial morphology revealed that the glutamate-induced mitochondrial fragmentation was reduced after siRNA-mediated GRP75 knockdown (Figure 5a; Supplementary Figure 1) with siRNA-01 (si01) indicating that mitochondrial integrity was preserved through GRP75 silencing. Although GRP75 knockdown using si01 preserved mitochondrial morphology, quantification of the mitochondrial morphology in cells with reduced GRP75 levels as a consequence of siRNA-02 (si02) transfection did not reach statistical significance.

**Figure 6:**
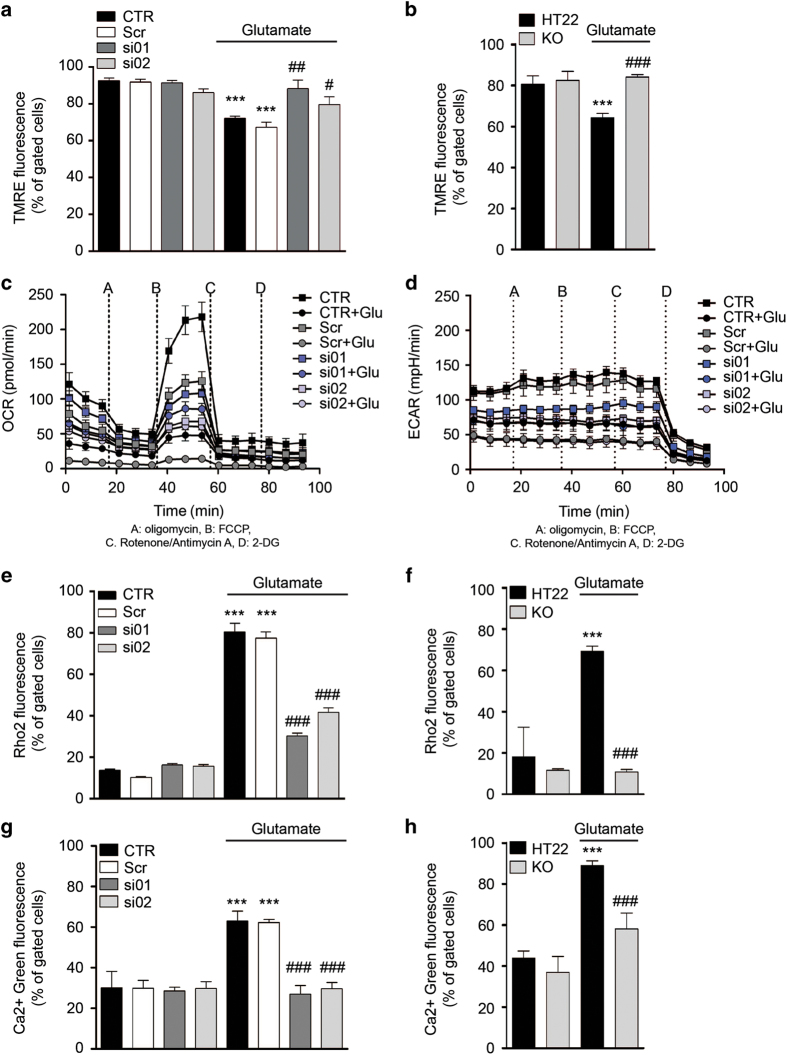
GRP75 knockdown and KO restores mitochondrial respiration and regulates Ca^2+^ flux following the glutamate challenge. (**a**, **b**) Representative measurement of the mitochondrial membrane potential following glutamate exposure (16 h) in (**a**) HT22 CTR and cells transfected with scrambled siRNA (Scr) or siRNA against GRP75 (si01, si02), and (**b**) HT22 and GRP75 KO cells. Data are presented as mean + S.D., *n*=3, ***P<0.0001 compared with control, ^#^*P*<0.05, ^##^*P*<0.01, ^###^*P*<0.0001 compared with glutamate. (**c**, **d**) Representative measurement of (**c**) oxygen consumption (OCR) and (**d**) extracellular acidification following glutamate exposure (16 h) in HT22 CTR and cells transfected with Scr or siRNA against GRP75 (si01, si02). Data are presented as mean ± S.D., *n*=6–8 per condition. (**e**, **f**) Representative measurement of mitochondrial Ca^2+^ following glutamate exposure (16 h) in (**e**) HT22 CTR and cells transfected with Scr or siRNA against GRP75 (si01, si02), and (**f**) HT22 and GRP75 KO cells. Data are presented as mean + S.D., *n*=3, ***P<0.0001 compared with control, ^###^*P*<0.0001 compared with glutamate. (**g**, **h**) Representative measurement of cytosolic Ca^2+^ following glutamate exposure (16 h) in (**g**) HT22 CTR and cells transfected with Scr or siRNA against GRP75 (si01, si02), and (**h**) HT22 and GRP75 KO cells. Data are presented as mean + S.D., *n*=3, ***P<0.0001 compared with control, ^###^*P*<0.0001 compared with glutamate. CTR, control cells.

Next, we investigated parameters indicating mitochondrial function in conditions of oxidative stress and altered GRP75 protein expression. ROS and the MMP^46^ were analyzed in response to glutamate using specific fluorescence-based probes and subsequent flow cytometric analysis. Glutamate-induced oxidative stress was featured by a substantial increase in mitochondrial ROS, and was attenuated in both, HT22 cells with reduced GRP75 expression (Figure 5b) and in GRP75 KO cells (Figure 5c). The MMP was disturbed following glutamate treatment which led to membrane depolarization. Both, RNA interference with GRP75 expression and KO were able to rescue the glutamate-induced loss of the mitochondrial membrane potential compared with the control cells (Figures 6a and b).

Furthermore, we analyzed the respiratory capacity of mitochondria (oxygen consumption rate; OCR) and the glycolytic activity (extracellular acidification rate; ECAR) after the glutamate challenge. Applying inhibitors of different respiratory chain complexes and 2-deoxyglucose to block glycolysis according to established protocols,^47,48^ allowed to evaluate OCR and ECAR. Glutamate reduced basal mitochondrial respiration, and attenuated the maximal respiration after uncoupling with FCCP compared with untreated control cells (Figure 6c). Interestingly, glutamate-challenged cells with reduced GRP75 expression showed higher basal and maximal respiration compared with glutamate-treated cells. In line with the OCR values, the ECAR values indicating glycolysis were also decreased in glutamate-treated cells. Silencing of GRP75 expression induced a decrease in the glycolytic activity compared with control cells, and partially restored it compared with glutamate-treated cells (Figure 6d). Similarly, inhibition of GRP75 by MKT-077 led to a decrease in basal and maximal respiration as well as to a decrease in glycolysis compared with control cells, yet to partial rescue of maximum respiration and the glycolytic capacity in conditions of glutamate-induced oxidative stress (Supplementary Figures 2a and b). Together, these results indicate a direct regulatory effect of GRP75 expression on energy metabolism in HT22 cells.

To prove that neuroprotection of HT22 cells by GRP75 silencing was mediated at the mitochondrial level and not upstream of mitochondrial damage at the level of ROS production, we investigated lipid peroxidation after 8 h of glutamate exposure which preceded mitochondrial damage (Supplementary Figure 2c). We found that GRP75 knockdown only partially blocked the glutamate-induced increase in lipid peroxidation while a substantial percentage of GRP75-silenced cells (60–70%) still showed enhanced lipid peroxidation. Thus, GRP75 knockdown-mediated neuroprotection against oxidative glutamate toxicity can be linked to its function at the mitochondria, likely at the level of ER–mitochondrial connections.

ER–mitochondrial junctions coordinate Ca^2+^ transfer from the ER into the mitochondria in forms of high local [Ca^2+^]_i_ microdomains.^9,21^ In order to investigate whether reduced ER–mitochondrial contact formation changed Ca^2+^ homeostasis, we determined cytosolic ([Ca^2+^]_c_) and mitochondrial ([Ca^2+^]_m_) calcium levels using specific fluorescence-based dyes. Fluorescence-activated cell sorting measurements revealed a massive glutamate-induced increase in [Ca^2+^]_m_ as detected by Rho2 staining which was abolished by both siRNAs against GRP75 (Figure 6e), and in the KO cells (Figure 6f). Furthermore, we analyzed changes in [Ca^2+^]_c_ by Ca^2+^ Green 5N staining. Glutamate treatment lead to elevated [Ca^2+^]_c_ levels which were attenuated by siRNA-mediated GRP75 knockdown (Figure 6g) and in KO cells (Figure 6h) compared with control cells.

In summary, GRP75 depletion provided protection against glutamate-induced oxidative stress by enhancing mitochondrial resilience. GRP75 silencing and KO restored mitochondrial function by maintaining the MMP, attenuating ROS formation, and by preserving the mitochondrial respiratory capacity. Notably, we found a major role for GRP75 in [Ca^2+^]_c_ and [Ca^2+^]_m_ handling during oxidative stress.

### Overexpression of GRP75 sensitizes HT22 cells to glutamate-induced cell death

**Figure 7:**
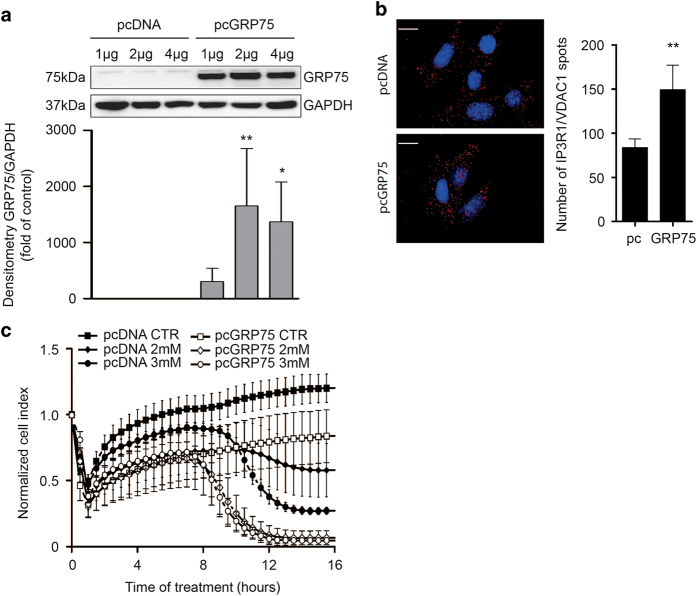
Overexpression of GRP75 enhances ER–mitochondrial coupling and sensitizes to oxytosis. (**a**) Overexpression of myc-tagged GRP75 in HT22 cells after transfection with different amounts of either pcDNA or pcGRP75 (1/2/4 *μ*g for 24 h). GAPDH is used as a loading control. GRP75 overexpression is quantified by densitometric analysis. Data are presented as mean + S.D., *n*=3, unpaired Student’s *t*-test, *P<0.05, **P<0.01. (**b**) *In situ* PLA in HT22 cells transfected with pcDNA or pcGRP75. Left panels: representative images after *in situ* PLA, DAPI-stained nuclei. Scale bar: 20 *μ*m. Right panel: quantification of IP_3_R/VDAC1 spots on the analyzed pictures. Data are presented as mean + S.E.M., *n*=25–30 per condition. (**c**) xCELLigence measurement of HT22 cells transfected with pcDNA or pcGRP75 following glutamate treatment (2 mM and 3 mM). Data are presented as mean ± S.D., *n*=6–8 per condition.

To further validate the impact of GRP75 expression on the ER–mitochondrial link in this paradigm of oxidative cell death, we overexpressed a myc-tagged plasmid encoding for GRP75 (pcGRP75) or a pcDNA3 control plasmid (pcDNA). Transfection with different plasmid amounts for 24 h resulted in an increase in c-myc-tagged GRP75 protein and mRNA expression after transfection with pcGRP75 compared with transfection with pcDNA alone (Figure 7a; Supplementary Figure 3a). In addition, *in situ* PLA analysis revealed that HT22 cells overexpressing GRP75 showed a higher number of IP_3_R1–VDAC1 interaction sites compared with cells overexpressing the control plasmid (Figure 7b). These results indicate that enhanced GRP75 expression tightens ER–mitochondrial coupling through increased ER–mitochondrial contact point formation.

Under physiological conditions, overexpression of either pcGRP75 or pcDNA did not reduce cell proliferation or cell survival as assessed by real-time impedance measurements (Supplementary Figure 3b). However, in conditions of oxidative glutamate toxicity GRP75 accelerated the susceptibility to cell death compared with pcDNA controls. Exposure of HT22 cells to glutamate reduced cell viability of pcDNA-transfected cells after ~10 h of treatment (Figure 7c). In contrast, the impedance in pcGRP75-transfected cells exposed to glutamate was already reduced 7.5 h after initiation of the damage. Notably, in response to glutamate, the cellular impedance of GRP75-transfected cells almost reached zero, indicating accelerated cell death, while a considerable portion of pcDNA-transfected cells was still viable.

Taken together, our results indicate that depletion of GRP75 provided protection against oxidative glutamate toxicity while pcGRP75 overexpression rendered HT22 cells more vulnerable to cell death and increased their sensitivity to oxidative stress through an increase in ER–mitochondrial contact formation.

## Discussion

Glutamate-induced oxidative cell death signaling culminates in the damage of the key organelles of energy metabolism, the mitochondria, which leads to irreversible cell death in HT22 cells. Therefore, mitoprotection is an emerging strategy to prevent neuronal cell death induced by oxidative stress. In the present study, we exploited the role of ER–mitochondrial contact formation in a model of glutamate-induced oxidative stress in immortalized neuronal HT22 cells. We found that relieving ER–mitochondrial coupling by genetic ablation of the chaperone GRP75 protected against oxidative glutamate toxicity by preserving mitochondrial morphology and function.

GRP75 is suggested to mediate organelle communication between the ER and mitochondria through interacting with both the IP_3_R and VDAC1.^10^ In this study, we performed an *in situ* proximity ligation assay between IP_3_R1 and VDAC1, and confirmed that GRP75 is a component of the complex that forms the MAM. In fact, we showed that GRP75 knockdown and pharmacological inhibition reduced the number of interaction sites between IP_3_R1 and VDAC1, thus reduced ER–mitochondrial coupling. In previous studies, GRP75 overexpression was associated with protection against cell death in cancer cells exposed to lethal cellular stress such as UV irradiation or in cultured astrocytes exposed to oxygen-glucose deprivation.^49,50^ Here, we showed that GRP75 was involved in pro-death rather than pro-survival signaling in neuronal HT22 cells. We found that glutamate-induced cell death was prevented in GRP75 knockdown and KO cells, whereas GRP75 overexpression enhanced the sensitivity of neuronal cells to the oxidative insult. Interestingly, the protective effect of GRP75 knockdown/KO was specific for oxidative glutamate toxicity as it failed to protect against ER stress. In line with our findings in the oxytosis model, GRP75 expression sensitized rat mesencephalic cells to rotenone toxicity.^28^ However, as opposed to mesencephalic cells, blocking GRP75 by MKT-077 in HT22 cells had no effect on rotenone-induced cell death regarding protection or higher vulnerability to cell death. Therefore, we conclude that the cellular function of GRP75 strongly depends on the cell type and the pathological context of how mitochondrial damage and cell death are initiated.

Inhibition of GRP75 with MKT-077 at low doses (50–200 nM) induced cell cycle arrest in a panel of human tumor cell lines.^25^ Furthermore, constant intraperitoneal injection of MKT-077 (3 mg/kg) inhibited tumor growth in a mouse liver cancer xenograft model and potentiated apoptosis of hepatocellular carcinoma cells induced by HSP90 inhibition.^51^ Both studies suggested a major role for GRP75 in regulating nucleo-cytoplasmic shuttling of the tumor suppressor p53. GRP75 is thought to bind to p53, thereby inhibiting p53 function by retaining it in the cytoplasm, and MKT-077 treatment allowed nuclear translocation of p53. Similarly, GRP75 downregulation by small hairpin RNAs or anti-peptides induced apoptosis in HepG2 liver cancer cells, and this effect was attributed to reduced GRP75–p53 interaction and concomitant nuclear p53 translocation in these cells.^52^ In contrast, in our study, MKT-077 reduced ER–mitochondrial contact points and conferred protection against oxidative glutamate toxicity suggesting the disconnection of ER and mitochondria as an underlying mechanism for the observed protection after GRP75 inhibition in HT22 cells. In favor of these findings, p53 was dispensable in the present model system.^53^ Thus, we conclude that ER–mitochondrial coupling facilitated by GRP75 has a critical role during oxidative stress in neuronal cells.

GRP75 was previously linked to mitochondrial protection, as knockdown or inhibition of GRP75 orthologs in yeast, *Caenorhabditis elegans* and *Drosophila melanogaster* induced mitochondrial damage.^54–56^ In line with these findings, inhibition of GRP75 was associated with neuronal disease pathology through mitochondrial fragmentation in a model of amyloid-*β* toxicity in glioblastoma cell lines.^57^ Moreover, GRP75 knockdown or overexpression of non-functional GRP75 mutants increased mitochondrial ROS formation and mediated mitochondrial membrane depolarization in HEK293 cells,^26^ and in HeLa cells exposed to H_2_O_2_.^58^ These previous findings showing deleterious effects of GRP75 inhibition are in contrast to our present findings in neuronal cells, where GRP75 depletion significantly blocked major hallmarks of glutamate toxicity that affected mitochondrial morphology and function, and cell death. Under physiological conditions, GRP75 knockdown did not change the mitochondrial morphology, whereas under conditions of oxidative stress, GRP75 depletion maintained the tubular shape and mitochondrial network in these cells. Further, GRP75 depletion did not produce mitochondrial ROS or depolarization of the mitochondrial membrane as observed in previous studies in non-neuronal cells, but rather restored these parameters to control levels following glutamate-induced stress. Interestingly, we found that reduced ER–mitochondrial coupling after GRP75 silencing decreased mitochondrial respiration compared with control cells independent of glutamate treatment, yet did not fully restore mitochondrial respiration back to control levels in glutamate-treated cells. These findings indicate that ER–mitochondrial coupling was important for the regulation of mitochondrial energy homeostasis which may contribute to the underlying protective mechanism independent of redox homeostasis or mitochondrial membrane integrity.

The capacity of mitochondria to buffer changes in [Ca^2+^]_i_ is essential to maintain [Ca^2+^]_i_ homeostasis. However, in HT22 cells oxidative stress induced massive [Ca^2+^]_m_ accumulation^59^ and enhanced ORAI1-dependent [Ca^2+^]_c_ entry.^60^ Here, we show that GRP75 ablation attenuated [Ca^2+^]_m_ overload following glutamate toxicity suggesting that oxidative stress was prevented by reduced Ca^2+^ transfer between ER and mitochondria. Interestingly, we found that GRP75 silencing also prevented glutamate-induced [Ca^2+^]_c_ entry. ORAI1-dependent extracellular Ca^2+^ entry is a late event during oxidative glutamate toxicity which is thought to refill intracellular Ca^2+^ stores.^60,61^ Therefore, we conclude that impairing the connectivity between ER and mitochondria by GRP75 silencing reduced ER-Ca^2+^ transfer into mitochondria which prevented mitochondrial damage, and in addition blocked late-stage Ca^2+^ dysregulation in the cytosol downstream of mitochondrial damage. The fact that siRNA-mediated downregulation of GRP75 did not prevent the glutamate-induced increase in lipid peroxidation, which occurs upstream of mitochondrial damage, further strengthens the concept that ER–mitochondrial coupling is important for mitochondrial integrity.

GRP75 overexpression *in vitro* prevented or delayed ROS accumulation in PC12 cells exposed to oxygen-glucose deprivation,^58,62,63^ in liver cells treated with H_2_O_2_^64^ and in a model of amyloid-*β* toxicity in SH-SY5Y cells.^65^ Moreover, GRP75 overexpression *in vivo* reduced the infarct size and protected from mitochondrial damage following focal ischemia induced by middle cerebral artery occlusion in rats.^66^ In sharp contrast to these protective effects of GRP75 overexpression, we show that GRP75 overexpression increased the susceptibility of HT22 cells to oxidative stress through enhanced contact formation between ER and mitochondria. In the current model of oxidative stress, these findings correspond to the fact that GRP75-depleted cells were resistant against the glutamate challenge. The discrepancy between our findings and the aforementioned studies might be attributed to the various cellular functions of GRP75 which seem to be different in different cell types. Besides its role in ER–mitochondrial coupling, GRP75 is also a major component of the mitochondrial import machinery, being identified as a regulator of MAPK (Akt/Erk)-dependent pro-survival signaling,^49,52^ and being linked to the regulation of p53. Moreover, the cell death or disease model seems to influence the outcome of GRP75 modulation. In our study, we identified a protective role for GRP75 knockdown in a model of oxidative stress initiated upstream of the mitochondria while cell death triggered directly at the level of the ER (brefeldin A and thapsigargin) or at the level of the mitochondria (rotenone) was not prevented. In addition, we did not observe an effect of GRP75 depletion in control conditions suggesting that glutamate enhanced the GRP75-dependent ER–mitochondrial coupling. Interfering with this physical link therefore provided protection against oxidative stress, while an additional tightening by GRP75 overexpression was even more harmful. These findings agree with an earlier study showing that enhanced ER–mitochondrial contact formation rendered cells more vulnerable to [Ca^2+^]_m_ overload, thereby facilitating the formation of the mitochondrial permeability transition pore (mPTP).^41^ In conditions where the cell death trigger ceased ER–mitochondrial coupling, thus reduces Ca^2+^ transfer rather than forcing it, GRP75 overexpression re-introduced the physical connection and restored Ca^2+^ signaling, thereby protecting against cell death.

ER stress induced by thapsigargin in human cancer cells^67^ or tunicamycin in HeLa cells^68^ promoted cell death involving mitochondrial swelling, dissipation of the electrochemical gradient and opening of the mPTP. However, in HT22 cells ER stress induced by brefeldin A, thapsigargin or tunicamycin induced caspase-dependent cell death through enhanced ER-Ca^2+^ release that was independent of mitochondrial damage.^48^ The fact that ER stress-mediated cell death was independent of mitochondrial performance might explain why GRP75 knockdown did not rescue the cells in the paradigm of ER stress. In line with our findings, GRP75 knockdown also reduced cell death induced by mitochondrial translocation of *α*-synuclein,^69^ and the authors argued that the disease state may determine protective *versus* detrimental effects of GRP75.

Similarly, contrasting effects were also described for other tethering proteins, such as mitofusin 2 (MFN2). MFN2 heterodimerizes with MFN1 to link ER and mitochondria, however, they also mediate mitochondrial fusion to drive mitochondrial metabolism.^70,71^ In rat skeletal muscle cells, MFN2 knockdown impaired mitochondrial fusion and induced oxidative stress in one study^72^ while MFN2 knockdown primarily decreased ER–mitochondrial coupling and reduced [Ca^2+^]_m_ in another study.^73^

In conclusion, we demonstrate that downregulation of GRP75 expression reduced ER–mitochondrial coupling and protected against oxidative glutamate toxicity in neuronal HT22 cells. The observed protection was mediated by attenuating ER-Ca^2+^ transfer to mitochondria which restored [Ca^2+^]_m_ homeostasis and enhanced mitochondrial resilience in conditions of oxidative stress. In turn, an increase in GRP75 expression increased the sensitivity of HT22 cells towards glutamate-induced oxidative cell death. Together, we report a protective function for GRP75 in the paradigm of oxytosis through the regulation of ER–mitochondrial coupling and related metabolic processes. To clarify the current discrepancies on GRP75 function in different cell types and paradigms of cellular stress, further studies are required to delineate the role of GRP75-mediated ER–mitochondrial contact formation and mitochondrial homeostasis in physiological and pathological conditions.

## Materials and methods

### Cell culture and plasmid transfection

HT22 cells were cultured in Dulbecco’s modified Eagle medium (DMEM; Sigma-Aldrich, Munich, Germany) supplemented with 10% heat-inactivated fetal calf serum (PAA, Cölbe, Germany), 100 U/ml penicillin, 100 *μ*g/ml streptomycin and 2 mM L-glutamine (Invitrogen, Karlsruhe, Germany) at 37 °C and 5% CO_2_.

HT22 cells were transfected with 1–4 *μ*g pcDNA or pcDNA-c-myc-GRP75 (pcGRP75) using the attractene transfection protocol (Qiagen, Hilden, Germany) for 24 h or 48 h depending on the plate format.

### RNA interference and reverse transcriptase PCR

For reverse transfection with siRNA, HT22 cells were seeded in antibiotic-free medium and transfected with non-specific universal negative siRNA (Sigma-Aldrich) and two different siRNAs directed against GRP75 (si01= ACACGGAGCAAUAGUUCUCUU, and si02= ACUUUAAGCUAUGGCUAACUU) using the lipofectamine RNAiMAXX transfection protocol (Thermo Fisher Scientific, Darmstadt, Germany) and incubated for 24 h. After overnight incubation, the cells were harvested, re-seeded into the desired culture plate format for subsequent experiments and incubated for further 24 h. For reverse transcriptase PCR, RNA was extracted from cell lysates after transfection with scrambled siRNA, si01 or si02 for 48 h using the InviTrap Spin Universal RNA Kit (Stratec Molecular, Berlin, Germany). For cDNA synthesis using the SuperScript III One-Step RT-PCR Kit with Platinum Taq (Invitrogen) and RT-PCR, 1 *μ*g RNA was used. To detect *Grp75* mRNA levels, the PCR was performed with the following protocol: 30 min 45 °C, 2 min 94 °C, 21×[15 s 94 °C, 30 s 50 °C, 45 s 68 °C], 5 min 68 °C using *Grp75* primers.

Forward primer 5′ TGCATCAGAAGCAATCAAGG 3′

Reverse primer 5′ TGGCCCAAGTAATTTTCTGC 3′

As a control, *Gapdh* mRNA was detected using the following PCR protocol: 30 min 60 °C, 2 min 95 °C, 21×[30 s 95 °C, 60 s 57 °C, 2 min 70 °C], 10 min 70 °C using *Gapdh* primers.

Forward primer 5′ CGTCTTCACCACCATGGAGAAGGC 3′

Reverse primer 5′ AAGGCCATGCCAGTGAGCTTCCC 3′.

PCR products were analyzed on a 1.5% agarose gel using UV illumination.

### CRISPR/Cas9-mediated GRP75 KO

GRP75 KO cells were generated using the CRISPR/Cas9 technique. HT22 cells were transfected for 48 h with a GFP-tagged CRISPR plasmid (pSpCas9_BB_2A-GFP (PX458); U6248BE310_1; GenScript, Piscataway, New Jersey, USA) with a specific gRNA against GRP75 and sorted for high GFP fluorescence, excluding dead cells via DAPI staining, and giving rise to one clonal colony (1-1; KO). GRP75 protein expression was controlled by western blot. Changes on DNA level were investigated by specific primers. To minimize the number of off target genes, the gRNA sequence was chosen using the CRISPR design database (crispr.mit.edu).

### Protein analysis and western blot

To analyze changes in protein expression, cell lysates were obtained by harvesting in lysis buffer containing 0.25 M mannitol, 0.05 M Tris-HCl, 1M EDTA, 1M EGTA, 1 mM DTT, 1% Triton X-100 and supplemented with Complete Mini Protease Inhibitor Cocktail and PhosSTOP (both Roche Diagnostics, Penzberg, Germany). Cell lysates were centrifuged at 10 000×*g* for 15 min at 4 °C to remove insoluble fragments. The total protein content was determined using the Pierce BCA Protein Assay Kit (Perbio Science, Bonn, Germany). For western blot analysis, 50 *μ*g of protein were loaded on a 10% SDS-gel and transferred onto a PVDF membrane. Incubation with the primary antibody was performed overnight at 4 °C. The following primary antibodies were used: rabbit polyclonal anti-GRP75 (Cell Signaling, Danvers, MA, USA), mouse monoclonal anti-c-myc 9E10 (Santa Cruz Biotechnology, Heidelberg, Germany), rabbit monoclonal anti-GADPH (Cell Signaling) and mouse monoclonal anti-vinculin (Sigma-Aldrich). Following overnight incubation, PVDF membranes were washed 3 times with 0.05% TBS-Tween and incubated with corresponding secondary HRP-labeled antibodies (Vector Laboratories, Burlingame, CA, USA). Protein expression was detected by chemiluminescence using the Chemidoc software (Bio-Rad, Munich, Germany) and quantified using the Quantity One software (Bio-Rad).

### *In situ* proximity ligation assay

ER–mitochondria interactions were analyzed using an optimized *in situ* proximity ligation assay (PLA) targeting the IP_3_R/GRP75/VDAC1 complex at the MAM interface, as previously described.^74,75^ Briefly, HT22 cells were cultured on 35 mm glass bottom dishes (MatTek, Ashland, MA, USA). After fixation with 4% paraformaldehyde for 10 min, the cells were permeabilized using 0.3% PBS/Triton X-100 for 30 min. Then, saturation was performed and *in situ* PLA experiments were done according to the manufacturer’s protocol. Briefly, VDAC1 (mouse anti-VDAC1 primary antibody) and IP_3_R1 (rabbit anti-IP3R1 primary antibody) were probed. Then, the secondary antibodies anti-mouse and anti-rabbit IgG (PLA probe MINUS and PLUS) conjugated to complementary oligonucleotide extensions were added. If the distance between the targeted proteins was below 40 nm, the oligonucleotides hybridized with the subsequently added connector oligonucleotides allowing the formation of a circular DNA template. This circular DNA molecule was ligated and amplified, thereby creating a single-stranded DNA product covalently attached to one of the proximity probes, and hybridized Texas red-labeled oligonucleotide probes were detected. Each fluorescent dot represents an interaction between VDAC1 and IP_3_R1. Preparations were mounted in Duolink II mounting medium containing DAPI 18 (Sigma-Aldrich) and analyzed with a Zeiss inversed fluorescent microscope at 63× magnification. Quantification of signals (number of red dots per cell) was done using the BlobFinder software. Experiments were performed in triplicate, *n*=10 pictures per conditions.

### Cell viability measurement

Cell viability was assessed in real-time using the xCELLigence system (Roche, Munich, Germany). HT22 cells were grown in 96well plates and treated with glutamate. Cellular impedance was measured every 30 min and represented as cellular index, normalized to the time of glutamate initiation. Alternatively, cell viability after glutamate exposure was assessed by addition of 3-(4,5-dimethylthiazol-2-yl)-2,5-diphenyltetrazolium bromide (MTT) at a final concentration of 0.5 mg/ml and incubation for 1 h at 37 °C. After removal of the medium from the plate and incubation at −80 °C for 1 h, the resulting purple formazan was dissolved in DMSO. Absorbance was measured at 570 nm *versus* 630 nm with FluoStar Optima (BMG Labtech, Offenbach, Germany). In order to exclude differences in basal cell viability while analyzing protection, the glutamate treatment for each condition was normalized to the corresponding control. In addition, cell death was analyzed by annexin V (early apoptosis) and propidium iodide (late apoptosis) double staining using the annexin-V-FITC detection kit (Promokine, Heidelberg, Germany) followed by flow cytometric analysis with excitation at 488 nm and detection 530 nm (green) and 680 nm (red). Data were recorded from 1×10^4^ cells in triplicate per condition.

### Mitochondrial morphology

HT22 cells were seeded into an iBidi containing 1.6×10^4^ cells per well and grown overnight. Before initiating the glutamate challenge with 8–10 mM glutamate for 16 h, mitochondria were stained with MitoTracker Green FM (Thermo Fisher Scientific) and DAPI for 30 min at 37 °C. After glutamate treatment, the plate was washed with PBS and cells were fixed with 4% paraformaldehyde for 25 min at room temperature. Images were acquired with a Leica epifluorescence microscope using a 63× magnification objective and additional 1.6× magnification. A total of ~300 cells per condition were counted. Mitochondria were classified into category I (elongated, distributed throughout the whole cell), category II (elongated and partially fragmented) or category III (fragmented, accumulated around the nucleus). Images were analyzed using the ImageJ software (Wayne Rasband, National Institutes of Health, Bethesda, MD, USA).

### Mitochondrial Ca^2+^ overload

Changes in mitochondrial Ca^2+^ were determined by the rhodamine-2-acetoxymethylester dye (Rho2-AM, Life Technologies, Carlsbad, CA, USA). Cells were harvested and incubated with 2 *μ*M dye in DMEM without serum for 25–30 min followed by incubation in DMEM for 25–30 min at room temperature in the dark. The fluorescence was excited at 552 nm and detected at 581 nm using the Guava Easy Cite 6-2L system (Merck Millipore, Darmstadt, Germany). Data were recorded from 1×10^4^ cells in triplicate per condition.

### Cytosolic Ca^2+^ entry

Changes in cytosolic Ca^2+^ were determined by a Ca^2+^ dye (Ca^2+^ Green 5N, Life Technologies). Cells were harvested and incubated with 2 *μ*M dye for 30 min at room temperature. The fluorescence was excited at 506 nm and detected at 532 nm using the Guava Easy Cite 6-2L system (Merck Millipore). Data were recorded from 1×10^4^ cells in triplicate per condition.

### Mitochondrial superoxide (ROS) formation

Mitochondrial ROS formation was assessed by the MitoSOX dye (Invitrogen). Cells were incubated with 2.5 *μ*M MitoSOX dye for 30 min at 37 °C and harvested afterwards. Fluorescence was excited at 488 nm and detected at 690/50 nm. Data were recorded from 1×10^4^ cells in triplicate per condition.

### Measurement of the mitochondrial membrane potential (Δ*ψ*_m_)

Loss of the Δ*ψ*_m_ was evaluated by staining with TMRE (tetramethylrhodamine-ethyl ester; Invitrogen) dye. Cells were harvested and incubated 30 min with 0.2 *μ*M TMRE at 37 °C. TMRE fluorescence was excited at 488 nm and detected at 690/50 nm. Data were recorded from 1×10^4^ cells in triplicate per condition.

### Seahorse XF analysis

HT22 cells were grown and treated with glutamate in Seahorse XF 96-well plates (Seahorse Biosystems, Agilent Technolgies, Waldbronn, Germany). Before the measurement, the medium was removed and replaced by 180 μl assay medium containing 4.5 g/l glucose, 2 mM L-glutamine, 1 mM pyruvate (pH 7.35) for 1 h at 37 °C. Using the Seahorse XF Biosystem, OCR and ECAR were analyzed. Three baseline measurements were recorded followed by 4 different injections: 3 *μ*M oligomycin in port A (20 *μ*l), 0.5 *μ*M FCCP in port B (22.5 *μ*l), 100 nM rotenone and 1 *μ*M antimycin A in port C (25 *μ*l), and 50 mM 2-deoxyglucose in port D (27.5 *μ*l). After injection of each compound, OCR and ECAR were measured (3 min mix and 3 min measure).

### Lipid peroxidation

Lipid peroxidation after 8 h of glutamate exposure was analyzed by staining with 2 *μ*M BODIPY dye (Invitrogen) for 60 min at 37 °C. A shift in BODIPY fluorescence from red to green was assessed by excitation at 488 nm and detection with a 525/30 nm band pass filter and a 690/50 nm band pass filter. Data were recorded from 1×10^4^ cells in triplicate per condition.

### Statistical analysis

Statistical significance was assessed using the unpaired Student’s *t*-test or ANOVA and Scheffé’s test for multiple comparisons, unless otherwise stated. *P*-values indicating statistically significant differences between the mean values are defined as follows: *P<0.05, **P<0.01 and ***P<0.001.

## Additional information

**Publisher’s note**: Springer Nature remains neutral with regard to jurisdictional claims in published maps and institutional affiliations.
