# Supplementary figures and images for: Glucose-regulated protein 75 determines ER–mitochondrial coupling and sensitivity to oxidative stress in neuronal cells
Source: Cell Death Discov. 2017 Nov 6;3:17076–. doi: 10.1038/cddiscovery.2017.76 (PMC5672593; doi:10.1038/cddiscovery.2017.76)

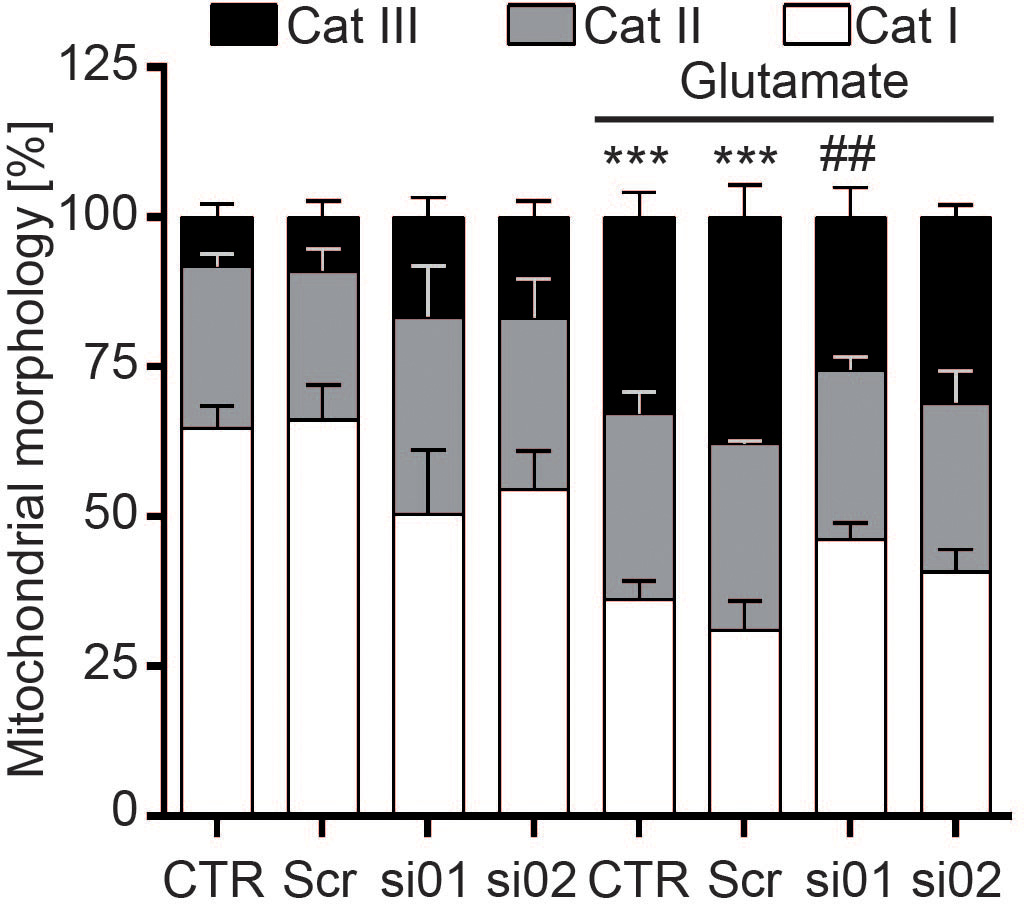

Supplement: Supplementary Figure 1 [file cddiscovery201776-s1.jpg]

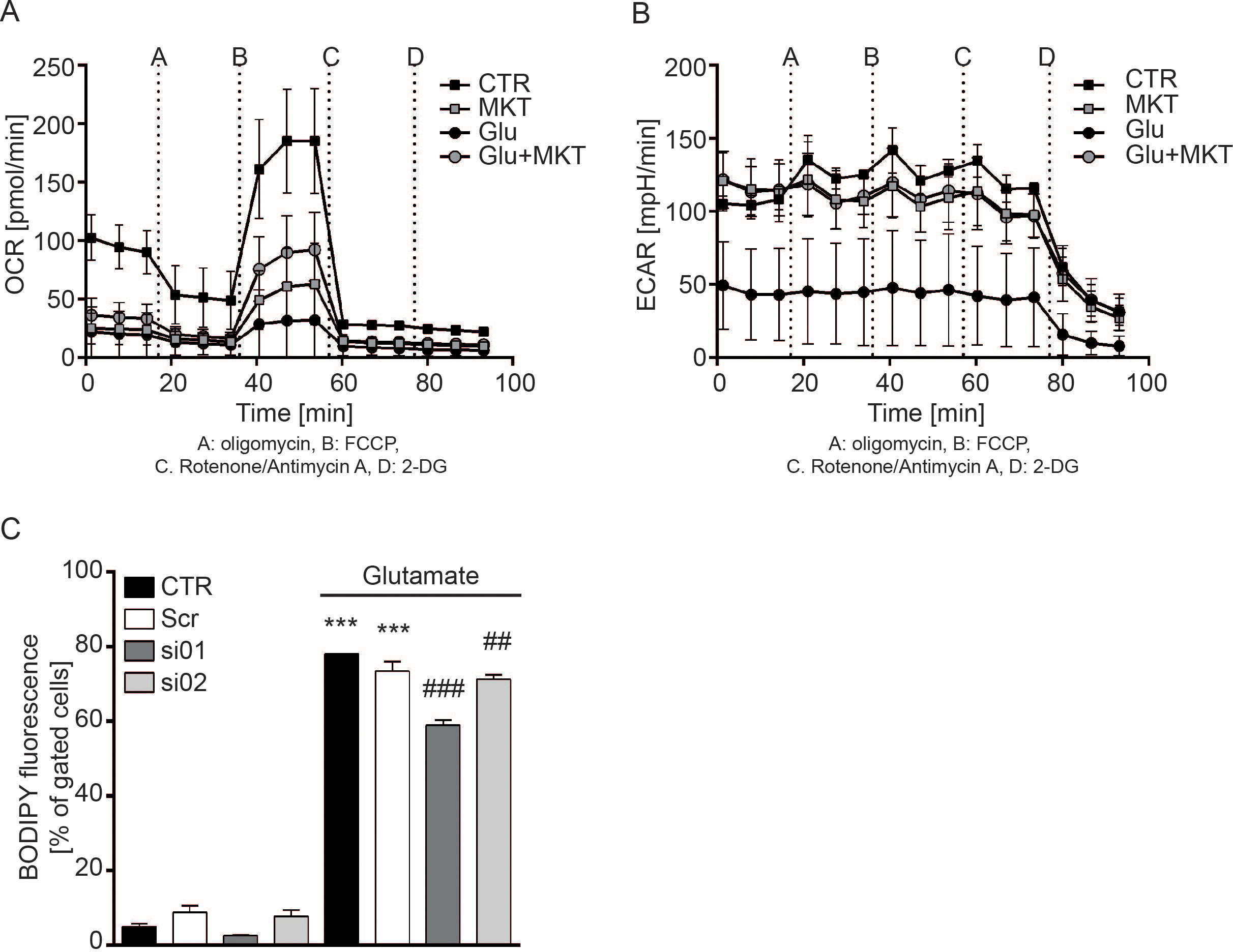

Supplement: Supplementary Figure 2 [file cddiscovery201776-s2.jpg]

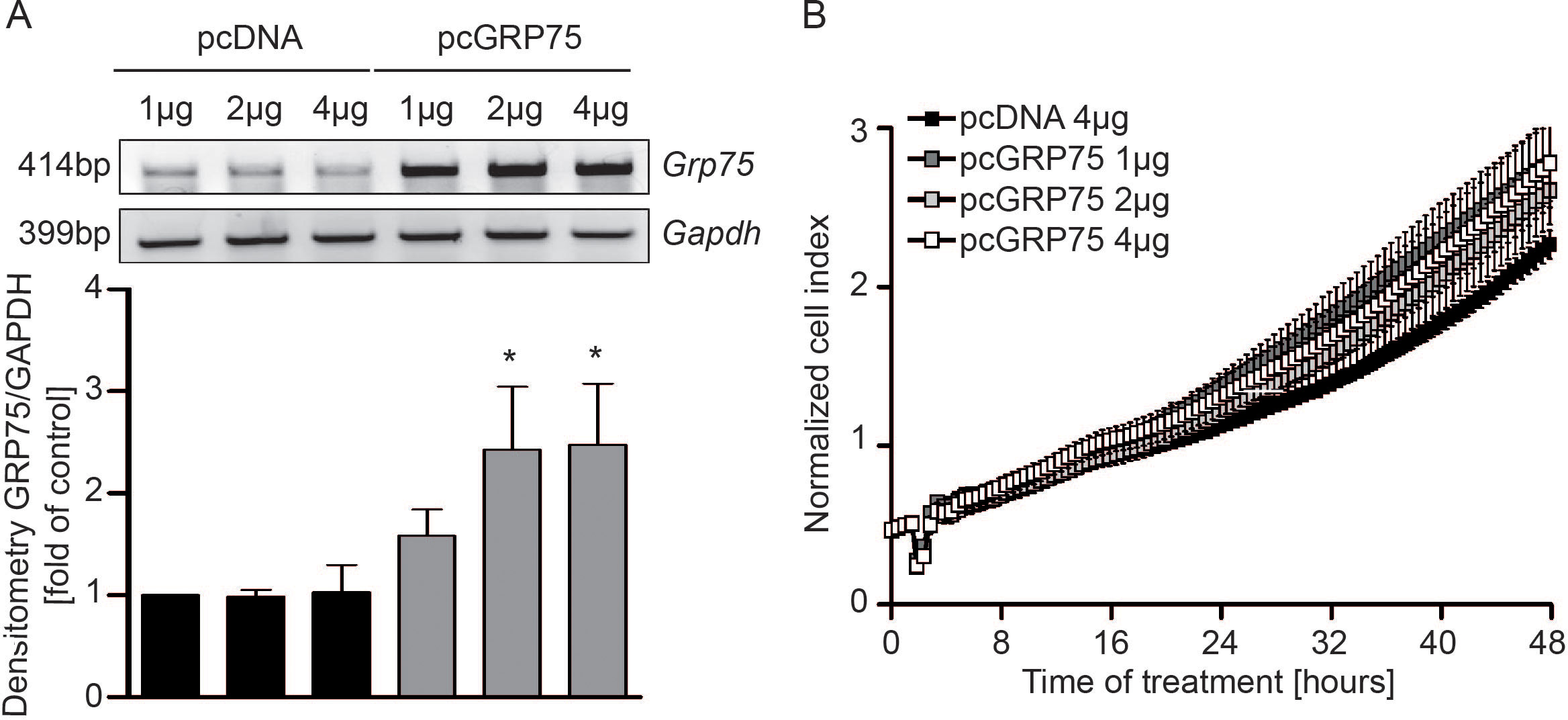

Supplement: Supplementary Figure 3 [file cddiscovery201776-s3.jpg]
