# Supplementary material for: Glucose-regulated protein 75 determines ER–mitochondrial coupling and sensitivity to oxidative stress in neuronal cells
Source: Cell Death Discov. 2017 Nov 6;3:17076–. doi: 10.1038/cddiscovery.2017.76 (PMC5672593; doi:10.1038/cddiscovery.2017.76)
Supplement: Supplementary Figure Legends [file cddiscovery201776-s4.docx]

## Supplementary figure 1. GRP75 knockdown preserves mitochondrial morphology.

Quantification of mitochondrial morphology analysis HT22 control cells (CTR) and cells transfected with scrambled siRNA (Scr) or siRNA against GRP75 (si01, si02). Mitochondria are categorized into category I (elongated), category II (elongated and fragmented) or category III (fragmented). Data are presented as mean + SD, 300 cells/condition, n=3, unpaired Student’s t-test, ***p<0.0001 compared to control, ^##^p<0.01 compared to glutamate.

## Supplementary figure 2. Effect of GRP75 inhibition/depletion on mitochondrial respiration and lipid peroxidation.

(a, b) Representative measurement of (a) oxygen consumption (OCR) and (b) glycolysis (extracellular acidification, ECAR) following glutamate exposure (16h) in HT22 cells treated with 10µM MKT-077. Data are presented as mean ± SD, n=6-8 per condition.

(c) Representative measurement of the lipid peroxidation following glutamate exposure (16h) in HT22 control cells (CTR) and cells transfected with scrambled siRNA (Scr) or siRNA against GRP75 (si01, si02). Data are presented as mean + SD, n=3, ***p<0.0001 compared to control, ^###^p<0.0001 compared to glutamate.

## Supplementary figure 3. Overexpression of GRP75 does not affect proliferation in the absence of glutamate.

(a) Detection of *Grp75* and *Gapdh* mRNA levels in HT22 cells after transfection with different amounts of either pcDNA or pcGRP75 (1/2/4µg for 24h). GRP75 overexpression is quantified by densitometric analysis. Data are presented as mean + SD, n=4, unpaired Student’s t-test, *p<0.05.

(b) xCELLigence measurement of HT22 cells transfected with pcDNA (4µg) or pcGRP75 (1/2/4µg). Data are presented as mean ± SD, n=6-8 per condition.
